# 4-Phenyl­semicarbazide

**DOI:** 10.1107/S1600536809035284

**Published:** 2009-09-05

**Authors:** Uzma Ashiq, Rifat Ara Jamal, Muhammad Nadeem Arshad, Zahida Tasneem Maqsood, Islam Ullah Khan

**Affiliations:** a Department of Chemistry, University of Karachi, Karachi 75270, Pakistan; b Department of Chemistry, Government College University, Lahore, Pakistan

## Abstract

The title compound, C_7_H_9_N_3_O, crystallizes with two independent mol­ecules per asymmetric unit. The structure is stabilized by four distinct inter­molecular N—H⋯O hydrogen bonds. Four intra­molecular inter­actions of the N—H⋯N and C—H⋯O types are also observed.

## Related literature

For related structures see: Ashiq, Jamal *et al.* (2008 ▶, 2009 ▶); Jamal *et al.* (2008 ▶ ▶, 2009 ▶); Kallel *et al.* (1992 ▶); Saraogi *et al.* (2002 ▶); For the biological activity of hydrazides, see: Ara *et al.* (2007 ▶); Ashiq, Ara *et al.* (2008 ▶).
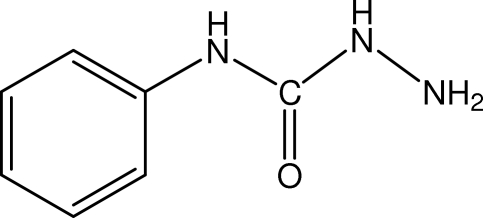


## Experimental

### 

#### Crystal data

C_7_H_9_N_3_O
*M* *_r_* = 151.17
Monoclinic, 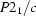

*a* = 16.5984 (10) Å
*b* = 8.8862 (4) Å
*c* = 10.3518 (6) Å
β = 91.359 (3)°
*V* = 1526.43 (14) Å^3^
*Z* = 8
Mo *K*α radiation
μ = 0.09 mm^−1^
*T* = 296 K
0.43 × 0.15 × 0.12 mm

#### Data collection

Bruker Kappa APEXII CCD diffractometer
Absorption correction: multi-scan (*SADABS*; Bruker, 2005 ▶) *T*_min_ = 0.979, *T*_max_ = 0.990
15394 measured reflections
3500 independent reflections
2258 reflections with *I* > 2σ(*I*)
*R*_int_ = 0.030

#### Refinement

*R*[*F* ^2^ > 2σ(*F* ^2^)] = 0.039
*wR*(*F* ^2^) = 0.107
*S* = 1.03
3500 reflections
223 parameters
H atoms treated by a mixture of independent and constrained refinement
Δρ_max_ = 0.16 e Å^−3^
Δρ_min_ = −0.15 e Å^−3^

### 

Data collection: *APEX2* (Bruker, 2007 ▶); cell refinement: *SAINT* (Bruker, 2007 ▶); data reduction: *SAINT*; program(s) used to solve structure: *SHELXS97* (Sheldrick, 2008 ▶); program(s) used to refine structure: *SHELXL97* (Sheldrick, 2008 ▶); molecular graphics: *ORTEP-3 for Windows* (Farrugia, 1997 ▶) and *Mercury* (Macrae *et al.*, 2006 ▶); software used to prepare material for publication: *SHELXL97*.

## Supplementary Material

Crystal structure: contains datablocks I, global. DOI: 10.1107/S1600536809035284/pk2182sup1.cif

Structure factors: contains datablocks I. DOI: 10.1107/S1600536809035284/pk2182Isup2.hkl

Additional supplementary materials:  crystallographic information; 3D view; checkCIF report

## Figures and Tables

**Table 1:** Hydrogen-bond geometry (Å, °)

*D*—H⋯*A*	*D*—H	H⋯*A*	*D*⋯*A*	*D*—H⋯*A*
N11—H11*N*⋯N13	0.849 (16)	2.130 (15)	2.6149 (19)	116.0 (13)
N12—H12*N*⋯O21^i^	0.872 (15)	2.071 (15)	2.9386 (15)	172.8 (14)
N13—H14*N*⋯O11^ii^	0.887 (18)	2.383 (17)	3.2149 (18)	156.1 (15)
N21—H21*N*⋯N23	0.852 (16)	2.130 (17)	2.6093 (19)	115.3 (14)
N22—H22*N*⋯O11^iii^	0.901 (16)	2.079 (17)	2.9784 (17)	175.9 (14)
N23—H23*N*⋯O21^iv^	0.919 (17)	2.203 (17)	3.0850 (18)	160.5 (15)
C12—H12⋯O11	0.93	2.33	2.9119 (18)	120
C22—H22⋯O21	0.93	2.46	2.9833 (18)	116

Symmetry codes: (i) 
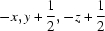
; (ii) 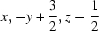; (iii) 
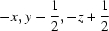
; (iv) 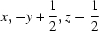.

**Fig. 1.**
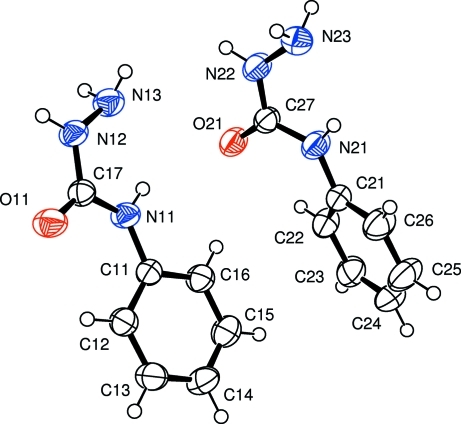
ORTEP diagram of the title compound with the ellipsoids drawn at the 50% probability level, showing the atomic labels.

**Fig. 2.**
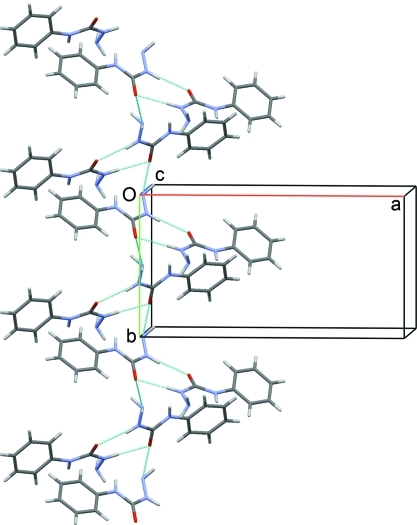
A packing diagram viewed down the c axis showing hydrogen bonds drawn as dashed lines. Hydrogen atoms not involved in H-bonding have been omitted.

## supplementary crystallographic information

### Comment

Hydrazides are known to have different biological activities (Ashiq, Ara *et al.*, 2008; Ara *et al.*, 2007). In order to study the biological activity of 4-phenylsemicarbazide, we undertook the synthesis of title compound and report its crystal structure in this paper. The title compound was found to be active against urease enzyme (Ara *et al.*, 2007). The structures of benzhydrazide (Kallel *et al.*, 1992), *para*-chloro (Saraogi *et al.*, 2002), *para*-bromo (Ashiq, Jamal *et al.*, 2008), *para*-iodo (Jamal *et al.*, 2008), *para*-methoxy (Ashiq, Jamal *et al.*, 2009) and *para*-hydroxy (Jamal *et al.*, 2009) analogues have already been reported.

The unit cell contains two crystallographically unique molecules, whose molecular structures are presented in Fig. 1. The bond distances and bond angles are similar to the corresponding distances and angles reported in the structures quoted above. The molecular packing diagram (Fig. 2) shows the presence of four intermolecular hydrogen bonds and four intramolecular hydrogen interactions.  In the crystal structure, two adjacent molecules are held together by intermolecular hydrogen bonds between the N12—H12N···O21, N13—H14N···O11, N22—H22N···O11 and N23—H23N···O21 (details are given in Table 1).

### Experimental

All reagent-grade chemicals were obtained from Aldrich and Sigma Chemical companies and were used without further purification. To a solution of phenyl urea (34 g, 0.25 moles) in 50 ml ethanol, hydrazine hydrate (25.0 ml, 0.45 moles) was added.  The mixture was refluxed for 24 h and solid 4-phenylsemicarbazide was obtained upon removal of the solvent by rotary evaporation (yield 79%).

### Refinement

H atoms were positioned geometrically, with C—H = 0.93 Å for aromatic C-atoms, and for N1 and N2 atoms were taken from fourier synthesis and their coordinates were refined with N—H = 0.849 (16)–0.918 (17)Å with U_iso_ set to 1.2U_eq_ of their parent atoms.

### Crystal data

**Table:**

C_7_H_9_N_3_O	*F*(000) = 640
*M**_r_* = 151.17	*D*_x_ = 1.316 Mg m^−^^3^
Monoclinic, *P*2_1_/*c*	Mo *K*α radiation, λ = 0.71073 Å
Hall symbol: -P 2ybc	Cell parameters from 3838 reflections
*a* = 16.5984 (10) Å	θ = 2.5–24.4°
*b* = 8.8862 (4) Å	µ = 0.09 mm^−^^1^
*c* = 10.3518 (6) Å	*T* = 296 K
β = 91.359 (3)°	Needle, colourless
*V* = 1526.43 (14) Å^3^	0.43 × 0.15 × 0.12 mm
*Z* = 8	

### Data collection

**Table:**

Bruker Kappa APEXII CCD diffractometer	3500 independent reflections
Radiation source: fine-focus sealed tube	2258 reflections with *I* > 2σ(*I*)
graphite	*R*_int_ = 0.030
ω scans	θ_max_ = 27.5°, θ_min_ = 2.5°
Absorption correction: multi-scan (*SADABS*; Bruker, 2005)	*h* = −21→20
*T*_min_ = 0.979, *T*_max_ = 0.990	*k* = −6→11
15394 measured reflections	*l* = −13→13

### Refinement

**Table:**

Refinement on *F*^2^	Primary atom site location: structure-invariant direct methods
Least-squares matrix: full	Secondary atom site location: difference Fourier map
*R*[*F*^2^ > 2σ(*F*^2^)] = 0.039	Hydrogen site location: inferred from neighbouring sites
*wR*(*F*^2^) = 0.107	H atoms treated by a mixture of independent and constrained refinement
*S* = 1.03	*w* = 1/[σ^2^(*F*_o_^2^) + (0.0459*P*)^2^ + 0.1614*P*] where *P* = (*F*_o_^2^ + 2*F*_c_^2^)/3
3500 reflections	(Δ/σ)_max_ < 0.001
223 parameters	Δρ_max_ = 0.16 e Å^−^^3^
0 restraints	Δρ_min_ = −0.15 e Å^−^^3^

### Special details

**Table:**

Geometry. Bond distances, angles *etc*. have been calculated using the rounded fractional coordinates. All su's are estimated from the variances of the (full) variance-covariance matrix. The cell e.s.d.'s are taken into account in the estimation of distances, angles and torsion angles
Refinement. Refinement of *F*^2^ against ALL reflections. The weighted *R*-factor *wR* and goodness of fit *S* are based on *F*^2^, conventional *R*-factors *R* are based on *F*, with *F* set to zero for negative *F*^2^. The threshold expression of *F*^2^ > σ(*F*^2^) is used only for calculating *R*-factors(gt) *etc*. and is not relevant to the choice of reflections for refinement. *R*-factors based on *F*^2^ are statistically about twice as large as those based on *F*, and *R*- factors based on ALL data will be even larger.

### Fractional atomic coordinates and isotropic or equivalent isotropic displacement parameters (Å^2^)

**Table:**

	*x*	*y*	*z*	*U*_iso_*/*U*_eq_	
O11	0.01604 (6)	0.79747 (12)	0.48141 (10)	0.0553 (4)	
N11	0.09359 (7)	0.58992 (13)	0.44101 (12)	0.0427 (4)	
N12	−0.01754 (7)	0.64373 (13)	0.31698 (11)	0.0438 (4)	
N13	−0.00041 (8)	0.51513 (16)	0.24513 (14)	0.0506 (5)	
C11	0.15892 (8)	0.60879 (14)	0.52831 (12)	0.0354 (4)	
C12	0.16095 (9)	0.71248 (16)	0.62779 (13)	0.0439 (5)	
C13	0.22909 (10)	0.72368 (18)	0.70707 (15)	0.0508 (5)	
C14	0.29449 (9)	0.63342 (19)	0.68932 (15)	0.0554 (6)	
C15	0.29205 (10)	0.52914 (19)	0.59133 (16)	0.0572 (6)	
C16	0.22502 (9)	0.51619 (17)	0.51125 (14)	0.0462 (5)	
C17	0.03011 (8)	0.68266 (15)	0.41816 (13)	0.0378 (5)	
O21	0.17516 (6)	0.28317 (12)	0.23819 (10)	0.0529 (4)	
N21	0.28009 (7)	0.40092 (15)	0.13966 (13)	0.0500 (5)	
N22	0.15623 (8)	0.38933 (15)	0.04272 (12)	0.0488 (4)	
N23	0.18846 (8)	0.47117 (17)	−0.05959 (13)	0.0514 (5)	
C21	0.34464 (8)	0.38042 (16)	0.22790 (14)	0.0433 (5)	
C22	0.34917 (9)	0.26384 (18)	0.31535 (15)	0.0509 (5)	
C23	0.41651 (9)	0.2490 (2)	0.39537 (16)	0.0593 (6)	
C24	0.47943 (10)	0.3476 (2)	0.38889 (17)	0.0643 (6)	
C25	0.47515 (10)	0.4626 (2)	0.30239 (18)	0.0707 (7)	
C26	0.40834 (9)	0.48014 (19)	0.22223 (17)	0.0596 (6)	
C27	0.20285 (8)	0.35311 (15)	0.14604 (14)	0.0405 (5)	
H11N	0.0960 (9)	0.5210 (18)	0.3845 (15)	0.0510*	
H12	0.11680	0.77440	0.64140	0.0530*	
H12N	−0.0624 (9)	0.6927 (17)	0.3025 (14)	0.0530*	
H13	0.23030	0.79400	0.77360	0.0610*	
H13N	−0.0413 (10)	0.4498 (19)	0.2569 (15)	0.0610*	
H14	0.34000	0.64240	0.74280	0.0660*	
H14N	0.0017 (10)	0.5393 (19)	0.1621 (17)	0.0610*	
H15	0.33610	0.46660	0.57900	0.0690*	
H16	0.22410	0.44500	0.44540	0.0550*	
H21N	0.2880 (10)	0.4551 (18)	0.0733 (16)	0.0600*	
H22	0.30700	0.19530	0.32060	0.0610*	
H22N	0.1043 (10)	0.3598 (17)	0.0395 (15)	0.0590*	
H23	0.41900	0.17040	0.45470	0.0710*	
H23N	0.1844 (10)	0.4143 (19)	−0.1336 (16)	0.0620*	
H24	0.52460	0.33620	0.44290	0.0770*	
H24N	0.1591 (10)	0.5550 (19)	−0.0679 (15)	0.0620*	
H25	0.51780	0.53010	0.29720	0.0850*	
H26	0.40610	0.55970	0.16390	0.0720*	

### Atomic displacement parameters (Å^2^)

**Table:**

	*U*^11^	*U*^22^	*U*^33^	*U*^12^	*U*^13^	*U*^23^
O11	0.0570 (7)	0.0548 (7)	0.0534 (7)	0.0192 (5)	−0.0156 (5)	−0.0142 (5)
N11	0.0424 (7)	0.0419 (7)	0.0434 (7)	0.0079 (6)	−0.0090 (6)	−0.0063 (5)
N12	0.0394 (7)	0.0465 (7)	0.0449 (7)	0.0100 (6)	−0.0088 (6)	−0.0061 (6)
N13	0.0484 (8)	0.0510 (8)	0.0519 (8)	0.0034 (6)	−0.0074 (7)	−0.0105 (7)
C11	0.0355 (7)	0.0362 (7)	0.0345 (7)	−0.0003 (6)	0.0002 (6)	0.0061 (6)
C12	0.0433 (8)	0.0468 (8)	0.0416 (8)	0.0066 (7)	−0.0014 (6)	−0.0018 (7)
C13	0.0546 (10)	0.0539 (9)	0.0435 (9)	−0.0006 (8)	−0.0050 (7)	−0.0052 (7)
C14	0.0438 (9)	0.0679 (11)	0.0537 (10)	0.0002 (8)	−0.0136 (7)	0.0009 (8)
C15	0.0435 (9)	0.0658 (11)	0.0619 (10)	0.0155 (8)	−0.0063 (8)	−0.0005 (8)
C16	0.0438 (8)	0.0480 (9)	0.0464 (9)	0.0087 (7)	−0.0042 (7)	−0.0025 (7)
C17	0.0372 (8)	0.0392 (8)	0.0370 (8)	0.0024 (6)	−0.0014 (6)	0.0032 (6)
O21	0.0386 (6)	0.0680 (7)	0.0520 (7)	−0.0068 (5)	−0.0024 (5)	0.0135 (5)
N21	0.0380 (7)	0.0630 (9)	0.0488 (8)	−0.0090 (6)	−0.0040 (6)	0.0102 (6)
N22	0.0372 (7)	0.0595 (8)	0.0495 (8)	−0.0050 (6)	−0.0050 (6)	0.0095 (6)
N23	0.0517 (8)	0.0591 (9)	0.0432 (8)	0.0001 (6)	−0.0026 (6)	0.0044 (7)
C21	0.0340 (8)	0.0526 (9)	0.0433 (8)	−0.0017 (6)	0.0008 (6)	−0.0063 (7)
C22	0.0384 (8)	0.0566 (10)	0.0578 (10)	−0.0019 (7)	0.0009 (7)	0.0013 (8)
C23	0.0455 (10)	0.0743 (12)	0.0579 (10)	0.0094 (8)	−0.0022 (8)	0.0068 (9)
C24	0.0405 (9)	0.0906 (13)	0.0612 (11)	0.0034 (9)	−0.0121 (8)	−0.0091 (10)
C25	0.0445 (10)	0.0823 (13)	0.0846 (13)	−0.0177 (9)	−0.0116 (9)	−0.0025 (11)
C26	0.0488 (10)	0.0628 (11)	0.0669 (11)	−0.0135 (8)	−0.0044 (8)	0.0043 (8)
C27	0.0355 (8)	0.0419 (8)	0.0440 (8)	−0.0013 (6)	−0.0016 (6)	−0.0033 (7)

### Geometric parameters (Å, °)

**Table:**

O11—C17	1.2375 (17)	C12—C13	1.385 (2)
O21—C27	1.2362 (17)	C13—C14	1.366 (2)
N11—C11	1.4051 (18)	C14—C15	1.374 (2)
N11—C17	1.3541 (18)	C15—C16	1.376 (2)
N12—N13	1.3964 (18)	C12—H12	0.9300
N12—C17	1.3427 (18)	C13—H13	0.9300
N11—H11N	0.849 (16)	C14—H14	0.9300
N12—H12N	0.872 (15)	C15—H15	0.9300
N13—H14N	0.887 (18)	C16—H16	0.9300
N13—H13N	0.904 (17)	C21—C22	1.377 (2)
N21—C21	1.4032 (19)	C21—C26	1.382 (2)
N21—C27	1.3537 (18)	C22—C23	1.382 (2)
N22—C27	1.3443 (19)	C23—C24	1.366 (2)
N22—N23	1.4013 (19)	C24—C25	1.360 (3)
N21—H21N	0.852 (16)	C25—C26	1.378 (2)
N22—H22N	0.901 (16)	C22—H22	0.9300
N23—H23N	0.919 (17)	C23—H23	0.9300
N23—H24N	0.893 (17)	C24—H24	0.9300
C11—C12	1.3817 (19)	C25—H25	0.9300
C11—C16	1.386 (2)	C26—H26	0.9300
			
C11—N11—C17	128.70 (12)	C13—C12—H12	120.00
N13—N12—C17	120.18 (12)	C14—C13—H13	119.00
C11—N11—H11N	118.8 (10)	C12—C13—H13	119.00
C17—N11—H11N	111.6 (10)	C15—C14—H14	120.00
C17—N12—H12N	119.4 (10)	C13—C14—H14	120.00
N13—N12—H12N	120.0 (10)	C16—C15—H15	120.00
N12—N13—H14N	109.3 (11)	C14—C15—H15	120.00
H13N—N13—H14N	109.5 (15)	C15—C16—H16	120.00
N12—N13—H13N	106.9 (11)	C11—C16—H16	120.00
C21—N21—C27	129.56 (13)	C22—C21—C26	118.72 (14)
N23—N22—C27	120.18 (12)	N21—C21—C22	123.69 (13)
C21—N21—H21N	117.9 (11)	N21—C21—C26	117.53 (13)
C27—N21—H21N	112.4 (11)	C21—C22—C23	119.79 (14)
C27—N22—H22N	119.5 (10)	C22—C23—C24	121.19 (16)
N23—N22—H22N	120.4 (10)	C23—C24—C25	119.10 (16)
H23N—N23—H24N	110.4 (15)	C24—C25—C26	120.69 (16)
N22—N23—H24N	106.8 (10)	C21—C26—C25	120.50 (16)
N22—N23—H23N	108.8 (11)	O21—C27—N21	124.42 (13)
C12—C11—C16	119.04 (13)	O21—C27—N22	121.12 (13)
N11—C11—C16	116.64 (12)	N21—C27—N22	114.46 (13)
N11—C11—C12	124.32 (12)	C21—C22—H22	120.00
C11—C12—C13	119.65 (14)	C23—C22—H22	120.00
C12—C13—C14	121.24 (14)	C22—C23—H23	119.00
C13—C14—C15	119.07 (15)	C24—C23—H23	119.00
C14—C15—C16	120.69 (15)	C23—C24—H24	120.00
C11—C16—C15	120.30 (14)	C25—C24—H24	120.00
N11—C17—N12	114.90 (12)	C24—C25—H25	120.00
O11—C17—N11	124.42 (13)	C26—C25—H25	120.00
O11—C17—N12	120.67 (12)	C21—C26—H26	120.00
C11—C12—H12	120.00	C25—C26—H26	120.00
			
C17—N11—C11—C12	14.9 (2)	C16—C11—C12—C13	1.0 (2)
C17—N11—C11—C16	−164.97 (14)	N11—C11—C16—C15	178.94 (13)
C11—N11—C17—O11	−6.6 (2)	C11—C12—C13—C14	−0.3 (2)
C11—N11—C17—N12	171.74 (13)	C12—C13—C14—C15	−0.4 (2)
N13—N12—C17—O11	179.06 (13)	C13—C14—C15—C16	0.5 (2)
N13—N12—C17—N11	0.66 (18)	C14—C15—C16—C11	0.2 (2)
C21—N21—C27—O21	2.2 (2)	N21—C21—C22—C23	177.50 (14)
C27—N21—C21—C22	24.3 (2)	C26—C21—C22—C23	0.2 (2)
C27—N21—C21—C26	−158.35 (15)	N21—C21—C26—C25	−177.24 (15)
C21—N21—C27—N22	−178.53 (14)	C22—C21—C26—C25	0.2 (2)
N23—N22—C27—N21	−0.5 (2)	C21—C22—C23—C24	−0.5 (2)
N23—N22—C27—O21	178.77 (13)	C22—C23—C24—C25	0.3 (3)
N11—C11—C12—C13	−178.84 (13)	C23—C24—C25—C26	0.1 (3)
C12—C11—C16—C15	−0.9 (2)	C24—C25—C26—C21	−0.4 (3)

### Hydrogen-bond geometry (Å, °)

**Table:**

*D*—H···*A*	*D*—H	H···*A*	*D*···*A*	*D*—H···*A*
N11—H11N···N13	0.849 (16)	2.130 (15)	2.6149 (19)	116.0 (13)
N12—H12N···O21^i^	0.872 (15)	2.071 (15)	2.9386 (15)	172.8 (14)
N13—H14N···O11^ii^	0.887 (18)	2.383 (17)	3.2149 (18)	156.1 (15)
N21—H21N···N23	0.852 (16)	2.130 (17)	2.6093 (19)	115.3 (14)
N22—H22N···O11^iii^	0.901 (16)	2.079 (17)	2.9784 (17)	175.9 (14)
N23—H23N···O21^iv^	0.919 (17)	2.203 (17)	3.0850 (18)	160.5 (15)
C12—H12···O11	0.93	2.33	2.9119 (18)	120
C22—H22···O21	0.93	2.46	2.9833 (18)	116

Symmetry codes: (i) −*x*, *y*+1/2, −*z*+1/2; (ii) *x*, −*y*+3/2, *z*−1/2; (iii) −*x*, *y*−1/2, −*z*+1/2; (iv) *x*, −*y*+1/2, *z*−1/2.
